# Findings from a comprehensive diarrhoea prevention and treatment programme in Lusaka, Zambia

**DOI:** 10.1186/s12889-016-3089-7

**Published:** 2016-06-06

**Authors:** Samuel Bosomprah, Lauren B. Beach, Laura K. Beres, Jonathan Newman, Kabwe Kapasa, Cheryl Rudd, Lungowe Njobvu, Brad Guffey, Sydney Hubbard, Karen Foo, Carolyn Bolton-Moore, Jeffrey Stringer, Roma Chilengi

**Affiliations:** Center for Infectious Disease Research Zambia, 5032 Great North Road, P.O. Box 34681, 10101 Lusaka, Zambia; Department of Biostatistics, School of Public Health, University of Ghana, Accra, Ghana; Department of International Health, Bloomberg School of Public Health, Johns Hopkins University, Baltimore, MD USA; Department of Obstetrics and Gynecology, University of North Carolina School of Medicine, Chapel Hill, NC USA

## Abstract

**Background:**

The Programme for the Awareness and Elimination of Diarrhoea (PAED) was a pilot comprehensive diarrhoea prevention and control programme aimed to reduce post-neonatal, all-cause under-five mortality by 15 % in Lusaka Province. Interventions included introduction of the rotavirus vaccine, improved clinical case management of diarrhoea, and a comprehensive community prevention and advocacy campaign on hand washing with soap, exclusive breastfeeding up to 6 months of age, and the use of ORS and Zinc. This study aimed to assess the impact of PAED on under-5 mortality.

**Methods:**

The study was a pre-post evaluation design. The Demographic and Health Survey style population-based two-stage approach was used to collect data at the beginning of the intervention and 3 years following the start of intervention implementation in Lusaka province. The primary outcome of interest was an all-cause, post-neonatal under-five mortality rate defined as the probability of dying after the 28th day and before the fifth birthday among children aged 1–59 months. The Kaplan-Meier time to event analysis was used to estimate the probability of death; multiplying this probability by 1000 to yield the post-neonatal mortality rate. Survival-time inverse probability weighting model was used to estimate Average Treatment Effect (ATE).

**Results:**

The percentage of children under age 5 who had diarrhoea in the last 2 weeks preceding the survey declined from 15.8 % (95 % CI: 15.2 %, 16.4 %) in 2012 to 12.7 % (95 % CI: 12.3 %, 13.2 %) in 2015. Over the same period, mortality in post-neonatal children under 5 years of age declined by 34 %, from an estimated rate of 29 deaths per 1000 live births (95 % CI: (26, 32) death per 1000 live births) to 19 deaths per 1000 live births (95 % CI: (16, 21) death per 1000 live births). When every child in the population of children aged 1–59 months is exposed to the intervention, the average time-to-death was estimated to be about 8 months more than when no child is exposed (ATE = 7.9; 95 % CI: 4.4,11.5; *P* < 0.001).

**Conclusion:**

Well-packaged diarrhoea preventive and treatment interventions delivered at the clinic and community-level could potentially reduce probability of death among children aged 1–59 months.

## Background

In 2015, the global number of deaths among children under the age of 5 years (U5) was estimated as 5.95 million of which 3.26 million occurred in children aged 1–59 months [1]. Diarrhoea remains the second-leading cause of death among post-neonatal, under-five children, with 558,000 estimated diarrhoeal deaths, globally [2]. Rotavirus infection is the largest single cause of diarrhoea in children under 5, particularly of the severe diarrhoeal cases resulting in dehydration that lead to hospitalisation and death [3].

In 2015 in Zambia, an estimated 39,000 live-born children died before their fifth birthday, of which 26,000 occurred among post-neonatal under-five children [1]. Approximately 16 % of Zambian children U5 experience episodes of diarrhoea and about 66 % of these cases involve at least one health facility visit for diarrhoea [4].

Although rotavirus-associated diarrhoea can be prevented with rotavirus vaccine and can be treated with low-osmolarity oral rehydration salt (ORS) and zinc, a lack of awareness of effective strategies and preparedness for diarrhoea prevention and management leads to late presentation of ill children to the clinic and poor adherence to prescribed interventions. Efficacy trials of rotavirus vaccine have shown significant regional variability [5–8], and effectiveness data on comprehensive diarrhoea programmes in sub-Saharan Africa are largely unavailable.

In response, the Centre for Infectious Disease Research in Zambia (CIDRZ) and the UK-based charity Ark, in collaboration with the Zambian Ministry of Health (MOH) and other international stakeholders, developed and rolled out the Programme for the Awareness and Elimination of Diarrhoea (PAED), a demonstration pilot of comprehensive diarrhoea control within Lusaka Province in 2012. The goal of the PAED programme was to reduce all-cause U5 mortality by 15 %, as has been described elsewhere [9]. We hypothesised that an increase in effective diarrhoea prevention and treatment intervention coverage would decrease diarrhoea-associated morbidity and in turn decrease diarrhoea-associated mortality. The projected intervention impact was derived using the Lives Saved Tool (*LiST*) [10] under a number of assumptions on intervention components of the PAED programme including: rotavirus vaccination – from 0 to 90 % coverage with a 24 % mortality reduction; hand washing with soap – from 20 to 30 % coverage with a 48 % mortality reduction; exclusive breastfeeding promotion – from 15 to 35 to 65 % coverage and 3.5 fold risk reduction; and low-osmolarity ORS – from 53 to 75 % coverage with a 93 % mortality reduction; and zinc – from <5 to 40 % coverage with a 23 % mortality reduction [9]. The PAED was implemented from January 2012 – October 2014.

We elected to target post-neonatal mortality reduction because our interventions promise little or no effect to reduce neonatal mortality. Mortality reduction in the neonatal age-band has lagged behind gains achieved in the general under-5 population; about 44 % of all under-5 mortality happens within the first 28 days; this proportion is increasing as child deaths in the post neonatal age-band reduce [1, 11, 12].

In this paper, we present the evaluation of the programme through two rounds of household population-based surveys undertaken 3 years apart.

## Methods

### Study setting

At the time of the baseline study, Lusaka Province had four geographical districts: Lusaka, Kafue, Chongwe, and Luangwa. The four districts cover an area of 21,896 km^2^ and an estimated population of nearly 2.2 million [13]. There are just under 85,000 births a year in this province and more than 320,000 children under 5 years of age [13].

### Intervention

The PAED began in the four districts of Lusaka Province in early 2012. The PAED was designed to focus on three key project areas: i) rotavirus vaccination of approximately 180,000 infants, ii) a comprehensive community prevention and advocacy campaign, and iii) hands-on, procedural training of 560 first-responder clinicians in all 100 government health centres within the province. PAED was fully integrated into the government’s Expanded Programme on Immunisations and supported the establishment of cold chain, logistics and systems monitoring (e.g. vaccine and supply chain management) for the delivery of vaccines. The community prevention campaign communicated the benefits of hand washing with soap, and exclusive breastfeeding for diarrhoea prevention, as well as advocated for the rotavirus vaccine, and improved case management using low-osmolarity ORS and zinc for diarrhoea treatment.

### Sample size consideration

A post-neonatal under-five mortality rate of 23 deaths per 1000 live births was assumed for Lusaka Province based on the 2008 Zambia demographic health survey data. For each round of survey, a total of 21,956 households were required at 10 % precision and an estimated 17.2 % of under-five children constituting the Zambian population for an average household size of 5 with a nonresponse rate of 0.8 % and a design effect of 1.15. The national Central Statistical Office (CSO) has administratively divided the country into Census Supervisory Areas (CSAs). For a decision of 100 households to be selected per CSA, this translated into 220 CSAs selected in each round of survey. At baseline 100 households per CSA were sampled, while at endline, 125 households per CSA were sampled.

### Evaluation design

The study was a pre-post evaluation design. Baseline data on primary outcome and potential confounders were collected at the beginning of the programme in 2012. Endline data was collected 3 years following the implementation of the intervention in 2015 using similar survey design.

### Samples

A two-stage stratified random sampling design was used to sample households. Lusaka Province was stratified in 4 districts. The first stage was the ‘probability proportional to size’ selection of the CSAs in each district (stratum). The households in the selected CSAs constituted the secondary sampling units in the second stage of the sampling design. The allocation of CSAs was based on population density to include 80 % of the CSAs from the predominantly urban Lusaka District and 20 % of the CSAs from the other three more rural districts. A household was defined as a group of one or more related or unrelated people who share the same cooking and eating facilities and have one person who is regarded as the head of household, consistent with the CSO definition [4].

Surveyors sampled households by canvassing the CSA to visually identify the CSA boundaries defined by maps obtained from the CSO. They would then determine the approximate centre of the CSA and use a “spin of a bottle” method to determine the initial sampling direction. They approached every third household using a spiral or linear (spokes of a wheel) approach, depending on the household density in the CSA. When surveyors arrived at a sampled household, they asked for the person who normally makes day-to-day decisions regarding care of the children and the household. This ‘manager’ of the household could be either male or female, but priority was given to females, as they are generally believed to be more knowledgeable about the health status of all family members. The household manager was eligible to enrol their household if they signed/thumb printed an informed consent form and were either an adult 18 years or older, or were a young woman 15–17 years who was married, pregnant, or had been pregnant. If no head of household was available, the interviewer returned up to three more times. These return visits usually took place either the same day or the following day. If follow-up visits remained unsuccessful, an eligible household that was most near the household was selected as a replacement household. The Informed Consent Forms (ICFs) were multi-lingual and written in four major Zambian languages. In cases where a potential participant was illiterate an impartial literate witness was engaged.

### Data collection

Data collection tools were adapted from a set of standard DHS programme questionnaires that have been used in many countries worldwide (http://dhsprogram.com/What-We-Do/Survey-Types/DHS-Questionnaires.cfm). A Household Questionnaire was used to collect information on characteristics of the household’s dwelling unit and characteristics of usual residents and visitors. It was also used to identify members of the household who were eligible for an individual interview. All eligible women aged 18–49 who had been members of the household for equal to or greater than 1 month were asked to provide a birth history and answer questions about their children using a women questionnaire. Women who were 15–17 years old, who were married, pregnant, or who had ever been pregnant and who had lived in the household for at least 1 month were also asked to provide a birth history. All women ever reporting pregnancy were included to avoid misclassification of stillbirth.

The survey questionnaires were programmed in an Open Data Kit (ODK) tablet platform and optimised for survey administration using Nexus 7 2013 tablets. Weekly quality assurance/quality control was performed to generate queries, which were sent to field staff to effect necessary corrections and feedback into the system.

### Definitions

To optimise objective collection of data, the following field definitions were applied:

- Diarrhoea: Reported 3 or more bouts of watery stools within a 24-hour period experienced within two weeks of the survey.
- Hand washing with soap: A mother reporting that soap is used when washing hands and the surveyors physically confirming availability of soap at the usual hand washing place within the household.
- ORS and zinc availability: Reported use of the drug and physical verification of the drug or sachet availability within the house at the time of the interview.

Rotavirus vaccination: Reported eligible infants in the household immunised and the surveyor verifying the record on the child’s under 5 card.

### Statistical analysis

The primary outcome of interest was a post-neonatal under-five mortality rate defined as the probability of dying after the 28th day and before the fifth birthday among children aged 1–59 months. Every child recorded in the complete birth history dataset who was born within 5 years preceding each round of survey was included. For each round of survey, the Kaplan-Meier failure method was used to estimate the probability of death; multiplying the probability of death by 1000 gives the post-neonatal mortality rate expressed as deaths per 1000 live births. A child becomes at risk of death after 28 days following birth. The log rank test was used to compare the survival curves for the two rounds of surveys. Birth history data is suitable because it recorded the three key variables of survival data: date of birth of child, date of death of child (or age at death), and event status - death or alive.

Diarrhoea prevalence was computed as the proportion of U5 children who had diarrhoea in the two weeks prior to the survey. Coverage of key intervention components such as rotavirus vaccination, hand washing with soap as well as availability of ORS and zinc in households were also estimated to determine the level of penetration of the intervention.

Since the evaluation design was a nonrandomised-controlled trial we elected survival-time inverse-probability weighting regression adjustment (IPWRA) model to estimate ‘Average Treatment Effect’ (ATE), adjusting for potential confounders. We specified the treatment variable (i.e. pre-post intervention) as a logit function of a measure of ‘penetration’ of key intervention components (i.e. availability of ORS, zinc in the household, and Hand-washing with soap); the time to censoring was modelled as a Weibul function of sex of child and maternal age, while the outcome model was specified as Weibul with sex of child, maternal age, and diarrhoea incidence as covariates. The treatment effects models are efficient in strengthening the results of observational studies by using the potential outcome (counterfactual) framework.

The model has three assumptions. First, it requires the potential outcomes to be independent of the treatment assignment after conditioning on the covariates. Given that the intervention was not randomly assigned we elected to ensure this assumption by conditioning on key confounding variables in the model. Second, it requires that each individual in the study area have sufficient positive probability of being assigned to each treatment group. We investigated this assumption using a diagnostic approach in which we estimated the probabilities of treatment (i.e. propensity score) and compared its balance between covariates. Third, the model also requires correct adjustment for censoring - we accounted for censoring in the estimation of the ATE using Weilbul specification in the IPWRA model.

The analyses were performed using Stata version 14 (StatCorp, College Station, Texas, USA).

## Results

**Fig. 1:**
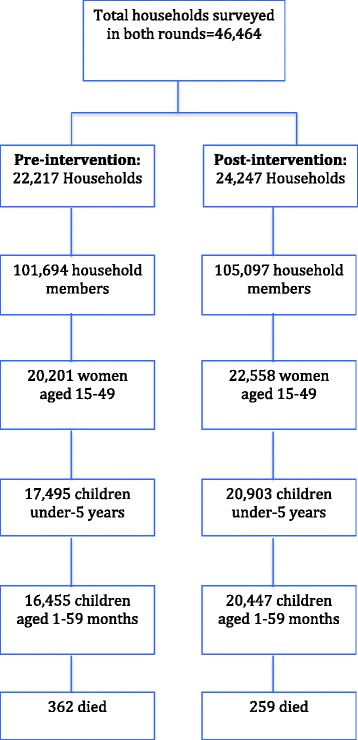
Flow of participants in the two rounds of surveys, 2012 to 2015, Lusaka Province, Zambia

A total of 46,464 households were surveyed in the two rounds of surveys: 22,217 at baseline, pre-intervention; and 24,247 at endline, post-intervention. These households accommodated 16,455 children aged 1–59 months at pre-intervention and 20,447 at post-intervention. Reported deaths among children aged 1–59 months were estimated as 362 in 2012 and 259 in 2015 (Fig. 1). The distribution of sex of child and mother’s age at birth was similar across intervention phases (Table 1).

**Table 1:** Background characteristics of children aged 1–59 months, Lusaka province, Zambia

Characteristics	Pre-intervention (2012)	Post-intervention (2015)	Total
	Number of children (% of total) *n* = 16,455	Number of children (% of total) *n* = 20,447	Number of children (% of total) *n* = 36,902
Sex of child			
Male	7,885 (47.9)	10,115 (49.5)	18,000 (48.8)
Female	8,135 (49.4)	10,332 (50.5)	18,467 (50.0)
Mother’s age at birth			
<20	2,930 (17.8)	3,960 (19.4)	6,890 (18.7)
20–34	11,988 (72.9)	14,599 (71.4)	26,587 (72.1)
35–49	1,448 (8.8)	1,838 (9.0)	3,286 (8.9)
Missing			
Districts			
Lusaka	13,428 (81.6)	14,864 (72.7)	28,292 (76.7)
Kafue	1,416 (8.6)	2,669 (13.1)	4,085 (11.1)
Chongwe	1,440 (8.8)	2,098 (10.3)	3,538 (9.6)
Luangwa	141 (0.9)	213 (1.0)	354 (1.0)
Diarrhoea prevalence	2,457 (15.8)	2,484 (12.7)	4,941 (14.2)

Between 2012 and 2015, hand washing with soap (soap visible to the interviewer) increased by about 19 %; availability of ORS in households for treating diarrhoea increased by about 66 %; and availability of zinc in households for treating diarrhoea increased by about 3 %; and in 2015 the coverage of under 5 card-confirmed rotavirus vaccination was estimated as 87 %; (Table 2).

**Table 2:** Coverage (%) of interventions in Lusaka province, Zambia, 2012–2015

Interventions	2012		2015		% increase from baseline
	Coverage (A)	95 % CI	Coverage (B)	95 % CI	[(B)-(A)]
Rotavirus vaccination (2 doses)^a^	-		86.7	[82.4,90.1]	-
Hand washing with soap (visible)	69.5	[66.9,72.1]	88.4	[87.0,89.7]	18.9
Low-osmolarity ORS	21.7	[19.4,24.1]	87.4	[86.0,88.7]	65.7
Zinc	7.8	[6.4,9.5]	11.2	[9.5,13.1]	3.4

^a^National rotavirus vaccination was introduced in November 2013, pilot introduction in January 2012

**Fig. 2:**
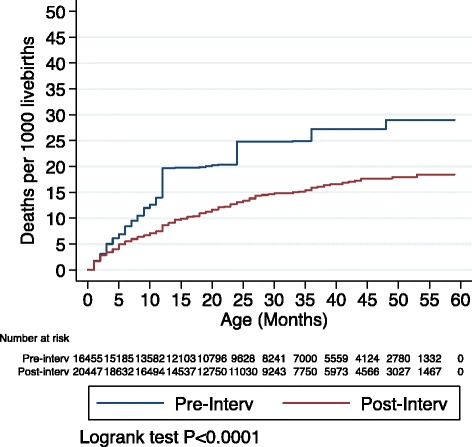
Kaplan Meier failure estimates (mortality per 1000 livebirths) Pre- Post-intervention among post-neonatal under-five children, Lusaka province, Zambia

The percentage of children under age 5 who had diarrhoea in the last 2 weeks preceding the survey declined from 15.8 % (95 % CI: 15.2 %, 16.4 %) in 2012 to 12.7 % (95 % CI: 12.3 %, 13.2 %) in 2015 (Table 1). Over the same period, mortality in post-neonatal children under 5 years of age declined by 34 %; from an estimated rate of 29 deaths per 1000 live births (95 % CI: [26, 32] death per 1000 live births) to 19 deaths per 1000 live births (95 % CI: [16, 21] death per 1000 live births) (Fig. 2). When every child in the population of children aged 1–59 months is exposed to the intervention, the average time-to-death was estimated to be about 8 months more than when no child is exposed (ATE = 7.9; 95 % CI: 4.4,11.5; *P* < 0.001) (Table 3).

**Table 3:** Average treatment effect (ATE) on time-to-death among children aged 1–59 months, 2012–2015, Lusaka Province, Zambia

Effects	Coefficient^a^	95 % CI	*P*-value
ATE			
Pre-intervention	ref		
Post-intervention	7.9	[4.4,11.5]	<0.001
Potential Outcome Mean			
Pre-intervention	9.4	[6.8,12.0]	<0.001
Relative ratio	0.85	[0.27,1.42]	<0.01

^a^Survival treatment effect estimation model (Estimator: inverse-probability weight regression adjustment; Outcome model: Weibul; Treatment model: logit; Censoring model: Weibul)

## Discussion

The results showed a 34 % reduction in the probability of death among U5 children who had survived beyond 1 month, over the period of implementation of PAED from 2012 to 2015. That is, in 2015, 1 in every 52 children born in Lusaka Province, who had survived the first 28 days, will die before attaining 5 years of age; as compared to 1 in every 34 children recorded at the start of the intervention. The results also showed that diarrhoeal prevalence declined by about 20 % over the same period.

Our findings are consistent with the general trend as reported in the Zambia Demographic and Health surveys from 85 post neonatal under-5 deaths per 1000 live births in 2007 to an estimated 51 deaths per 1000 live births in 2013 [4]. Given that the trends are similar and we did not have a contemporaneous control arm, we cannot attribute the mortality reduction to our intervention. Nonetheless, these results suggest that diarrhoea preventive interventions may contribute to substantial reduction in deaths among post-neonatal U5 children. Hand washing interventions have been shown to yield an estimated 32 % reduction in diarrhoea episodes in children living in low- or middle-income countries (IRR = 0.68, 95 % CI 0.52 to 0.90; 4 trials) [14, 15], whereas rotavirus vaccine prevented severe rotavirus episodes by about 50 % in sub-Saharan Africa [16]. Similarly, diarrhoea treatment interventions such as zinc and ORS have been estimated to reduce diarrhoea deaths by 23 % [10] and 93 % [17], respectively. In our study, we found that the coverage of the rotavirus vaccination among children aged 12–23 months was 87 %, while that of hand washing with soap and low osmolarity ORS were 88 and 87 % respectively. Use of zinc to treat diarrhoea was estimated to be about 11 % in this study. This is consistent with other findings that demonstrated that availability of zinc in Zambia was still poor with only about 10 % of the needed supply available in Zambia in 2012 [18].

Even with all the potential gains of the PAED interventions, there remain multiple gaps and challenges. Whereas the vaccination programme has been scaled-up nationally, vaccine coverage needs to be enhanced at national level for maximum population benefits. ORS and zinc coverage in the public sector remain low [18]. Taking the promotion of hand washing with soap and exclusive breastfeeding to national scale will also require well-funded dedicated programmes. Another gap based on the UNICEF-WHO recommended six-point strategy for diarrhoea control is access to safe drinking water and sanitation [19]. Upgrading and providing improved sanitation facilities for the 56 % of Zambians without access to improved sanitation facilities [4] could improve hygiene and thereby result in reduced diarrhoea episodes.

Nonetheless, our findings are very encouraging as they show a greater than double mortality reduction as planned by PAED (34 % vs. 15 %), acknowledging the downward trend nationwide. However, there are several limitations to these findings: We recognise methodological challenges limiting our temporal alignment between the behaviour change due to the intervention and post-neonatal U5 mortality. For example, the estimation of the intervention penetration was immediate – i.e. availability of ORS and zinc in the household, and hand washing with soap. A woman’s behaviour may not have changed when the baby died months before. As stated earlier, the best epidemiological design should have been a cluster randomized controlled (RCT) study design; but for national scale-up of proved interventions, we could not undertake a formal RCT. Second, the study was not powered to estimate the impact of the intervention in terms of diarrhoea cause-specific mortality. Lastly, by design our mortality data is based on recall by individuals, which has its own limitations [4, 20–22]. Given that this is not a controlled study, there may be some important biases - the lack of a control group means that history effects (extraneous events) and contemporaneous trends are an important threat to validity. Despite these limitations and challenges, there is evidence to suggest that targeting a major contributor to child deaths such as diarrhoea may yield important gains in reduced child mortality.

## Conclusion

Well-packaged diarrhoea preventive and treatment interventions delivered at the community and health facility levels may contribute to reductions in neonatal under-five mortality. Further efforts are required to take such impactful interventions to scale and sustain them.
